# Death from Encephalic Inflammation Consequent upon Aural Disease

**Published:** 1875-04

**Authors:** Swan M. Burnett

**Affiliations:** Knoxville, Tenn.


### DEATH FROM ENCEPHALIC INFLAMMATION CONSEQUENT UPON AURAL DISEASE.

BY SWAN M. BURNETT, M. D., KNOXVILLE, TENN.

On the first of July, 1874, I was asked by Dr. Boyd, the attending physician to the Tennessee School for the Deaf and Dumb at this place, to see, with him, one of the inmates who was suffering from a mastoidal inflammation.

I found a boy, aged 12, lying upon a bed in a semi-comotose condition, eyes closed, breathing heavy. With difficulty he could be made to heed our questions, put by way of signs, but soon lapsed again into the torpid state.

An almost unbearable stench came from his head, and filled the room, so that it was with difficulty one could remain near him for any length of time.

Examination revealed a redness and swelling of the right side of the head—most prominent behind the ear. There was tenderness on pressure, most marked in that region. The external meatus were so swollen that the membrana tympani could not be seen. An offensive discharge came from the ear. There was also a discharge of the same offensive character from the mouth.

From Dr. Boyd I learned the following history: Five days before, the patient had a chill followed by fever, and the next day the chill was repeated, accompanied by pain in the mastoid region, and some cerebral symptoms. An operation was then contemplated by Dr. Boyd, but the patient getting better next day, he thought he would be able to "tide" him over it without operative interference.

The next day, however, cerebral symptoms again making their appearance, he called me in with a view to opening the mastoid process, if found advisable.

It may be well to state, that the patient has been deaf since childhood, the deafness being the result of an inflammation of the ears at that time. Some years ago he had an attack similar to the present one, in the left ear, the remains of which are apparent in a large scar and depression on the mastoid region. There is still some discharge from the left ear; and ever since he has been in the institution he has suffered, at intervals, from pains in his ears.

Perforating the mastoid process was the only thing that offered any hope of relief, and I accordingly made an incision on a level with the superior wall of the meatus, and, with a trocar, perforated the wall of the mastoid. I found the wall thin, the bone rough; the cells broke down easily under the trocar. There was no immediate discharge of pus or other fluid; but on the next day, upon removing the clot of blood from the wound, there was a thin, dark, very offensive discharge, which kept up until death. The operation was not followed by any relief to the cerebral symptoms. He died on the 8th, with paralysis of the left side of the face and left arm. Unfortunately, no *post mortem* examination was obtained.

It is well known to aurists that encephalic inflammation is frequently the result of an inflammation of the middle ear and its appendages; but the fact is not sufficiently recognized by the general practitioner, under whose care these cases usually fall in their incipiency, when relief is most attainable.

Diseases of the mastoid form an interesting chapter in aural affections—but we have not here the space to enter into a consideration of them. The practitioner, however, should be made aware that inflammation of the mastoid process is likely to be followed by grave, if not fatal effects, and demands prompt interference. Whenever there is pain and swelling over the mastoid region, it is always well at once to make an incision down to the bone. This can result in no harm. Then, if the symptoms do not abate, perforate the wall and break down the cells. Even if there be no discharge, relief is almost certain.

The general practitioner need not be afraid of the operation, for it does not require more skill for its performance than a phlebotomy, and the effects of the operation are never more serious.

